# Insights into the interplay between Epstein‐Barr virus (EBV) and multiple sclerosis (MS): A state‐of‐the‐art review and implications for vaccine development

**DOI:** 10.1002/hsr2.1898

**Published:** 2024-02-15

**Authors:** Mahtab Mohammadzamani, Kimia Kazemzadeh, Swati Chand, Sangharsha Thapa, Narges Ebrahimi, Mohammad Yazdan Panah, Vahid Shaygannejad, Omid Mirmosayyeb

**Affiliations:** ^1^ Isfahan Neurosciences Research Center Isfahan University of Medical Sciences Isfahan Iran; ^2^ Students' Scientific Research Center Tehran University of Medical Sciences Tehran Iran; ^3^ Westchester Medical Center New York Medical College Valhalla New York USA; ^4^ Department of Neurology, Westchester Medical Center New York Medical College Valhalla USA; ^5^ Students Research Committee Shahrekord University of Medical Sciences Shahrekord Iran; ^6^ Department of Neurology Isfahan University of Medical Sciences Isfahan Iran

**Keywords:** EBV vaccination, Epstein‐Barr virus, HHV‐6, human herpesvirus 6, multiple sclerosis

## Abstract

**Background and Aims:**

Multiple sclerosis (MS) is a chronic autoimmune disease of the central nervous system (CNS). MS results from an inflammatory process leading to the loss of neural tissue and increased disability over time. The role of Epstein Barr Virus (EBV), as one of the most common global viruses, in MS development has been the subject of several studies. However, many related questions are still unanswered. This study aimed to review the connection between MS and EBV and provide a quick outline of MS prevention using EBV vaccination.

**Methods:**

For this narrative review, an extensive literature search using specific terms was conducted across online databases, including PubMed/Medline, Scopus, Web of Science, and Google Scholar, to identify pertinent studies.

**Results:**

Several studies proved that almost 100% of people with MS showed a history of EBV infection, and there was an association between high titers of EBV antibodies and an increased risk of MS development. Various hypotheses are proposed for how EBV may contribute to MS directly and indirectly: (1) Molecular Mimicry, (2) Mistaken Self, (3) Bystander Damage, and (4) Autoreactive B cells infected with EBV.

**Conclusion:**

Given the infectious nature of EBV and its ability to elude the immune system, EBV emerges as a strong candidate for being the underlying cause of MS. The development of an EBV vaccine holds promise for preventing MS; however, overcoming the challenge of creating a safe and efficacious vaccine presents a significant obstacle.

## 1 INTRODUCTION

Multiple sclerosis (MS) is a chronic autoimmune disorder of the central nervous system (CNS). In association with a compartmentalized inflammatory process, MS can cause neural tissue loss and progressive impairment.^1^ Globally, approximately 2.5 million individuals live with MS.^2^ The public health burden is substantial and encompasses both medical care costs and productivity losses due to the disease's impact on disability.^3^ This disorder's etiology is linked to various environmental and genetic factors. The environmental risk factors include obesity, smoking, lack of sun exposure leading to vitamin D deficiency, and Epstein‐Barr virus infection (EBV).^4^

In 1964, Epstein, Achong, and Barr first identified EBV as an Oncovirus with carcinogenic capabilities.^5^ The virus is highly prevalent in the population, leading to dormant infections that worsen over time and eventually exhibit immune‐modulating effects.^6^ According to a recent study, nearly 100% of MS patients with brain‐infiltrating B cells and plasma cells had evidence of EBV infection. In some instances, Ectopic B cell follicles found in the cerebral meninges were identified as the primary locations where EBV persists.^7^ EBV can play a significant role in MS development in different ways, such as (1) Cross‐reactivity between EBV and CNS antigens, bystander CNS damage caused by EBV‐specific CD8+ T cells, (2) activation of innate immunity by EBV‐encoded small RNA molecules in the CNS, and (3) infection of autoreactive B cells which produce harmful autoantibodies and send costimulatory survival signals to autoreactive T cells in the CNS.^8^ It has also been proposed that for a better understanding of MS pathophysiology as a chronic, autoimmune, demyelinating disease, myelin protein, and its relationship with EBV, it is better to analyze antibodies against native myelin oligodendrocyte glycoprotein.

Researchers assessed the gap between an infection and the development of MS in seronegative individuals to be 5.6 years based on serial serum samples available in a nested, case‐control study of MS patients with a history of primary EBV infection.^9^ As a result, it is fair to speculate that vaccination against infectious mononucleosis (IM) might lessen the incidence of MS. Importantly, it may establish (or refute) EBV as the causative agent of this illness.^10^

This study aimed to look at the possible links between EBV and MS and provide a concise summary of how MS might be prevented by getting vaccinated against EBV. In this review, we will carefully examine the pathophysiology of EBV disease and its relationship with MS and inflammatory and immunological pathways. We then discuss the history of the EBV vaccine and its ability to prevent MS.

## 2 EBV

EBV is a kind of human gamma‐herpesviruses with four subfamilies and a prototype of the genus Lymphocryptoviru.^11^ EBV infects over 95% of the human population and leads to IM in 70% of teenagers and young adults in developed nations.^12^ Both subtypes of EBV (EBV‐1 and EBV‐2) are commonly found in immunocompromised patients, and a pre‐existing immunosuppressive condition may be necessary for EBV‐2 to cause transformation. It has, however, become apparent that studies have challenged the notion that EBV‐2 superinfection is related to immunodeficiency.^13^ Kuri et al. surveyed 2325 individuals to determine the prevalence of anti‐EBV antibodies and followed them up. They reported that EBV seroprevalence in the United Kingdom and the incidence rate of IM requiring hospitalization seem to have increased.^14^ These findings clearly show the importance of EBV as a super prevalent virus and vaccination against EBV to prevent its following complications, including MS. To determine whether viral reactivation and impaired cell‐mediated immunity have occurred, biomarkers such as herpesvirus antibodies are used. Several studies indicated that members of the Black/African American community and Mexican Americans are more likely to be seropositive for herpes simplex virus 1 and 2 (HSV‐1 and HSV‐2), EBV, as well as for cytomegalovirus (CMV) than white individuals.^15^ Multiethnic studies have shown they can enhance our comprehension of MS vulnerability. The results of these studies indicated a significant biological connection between EBV infection and MS.^16^ It has been observed that while young African individuals exhibit higher levels of EBV antibodies, the incidence of MS in this demographic is lower compared to that in the other population. A plausible explanation for this phenomenon is that MS is a multifactorial disease.^17^ According to Maple et al. CMV infection can alter the immune response, potentially leading to a milder form of MS. Additionally, it may affect how the body responds to EBV infection.^18^

B cells become infected with EBV when the EBV envelope protein gp350 binds to complement receptor 2 (CR2)/CD21, expressed by mature B cells and follicular dendritic cells.^19^ Proliferating the lymphocytes by EBV leads to the activation of B cells, which then exploits the normal pathways of B‐cell differentiation into resting memory cells.^12^ B cells and epithelial cells require the EBV envelope proteins gH/gL and gB, whereas B cells require gp350 to infect effectively. The CD40 receptor assists in converting naive B cells into memory B cells when antigen stimulates them through the B‐cell receptor (BCR) and T‐cell assistance through the BCR.^20^ Incredibly, EBV indirectly activates BCRs and CD40 receptors in naive B cells while expressing two proteins: latent membrane protein 2A (LMP2A) and latent membrane protein 1 (LMP1).^21^ LMP1 and LMP2A are latency‐associated transcripts in latency II and III EBV‐infected cells.^22^ The immune system normally suppresses EBV by producing cytotoxic CD8+ T cells that destroy infected lymphocytes and promote the proliferation of normal B cells.^23^ Although the immune system can control EBV primary infection significantly, EBV remains a latent infection in B cells. During the productive phase of infection, CD4+ and CD8+ memory T cells can respond in a few hours. In this way, they can destroy the virus‐producing cells before the completion of viral replication and release of viral particles. Despite all this various range of immune mechanisms, EBV remains and persists for life by continuing to latent or lytic infection.^24^ Additionally, EBV can be treated as a target because it is a human virus latent in B cells only.^25^

## 3 EVIDENCE THAT EBV IS INVOLVED IN THE DEVELOPMENT OF MS

The prevalence of EBV seropositivity within MS patients has been extensively studied.^1^, ^26^ One new meta‐analysis has argued that 100% of MS patients are seropositive. The studies that found less have used a single method for detecting EBV, which means that a method for determining EBV serostatus highly affects the rate of EBV positivity in the population.^27^ Jacobs et al. suggest that although MS results from multiple risk modifiers, EBV is an obligate step in MS development.^3^ A meta‐analysis was conducted on seronegative MS patients. OR for developing adult MS in EBV was 0.18. following a sub‐group analysis, they found that pooled OR in the immunofluorescence method was 0.07, and in ELISA‐based studies, it was 0.33.^27^ Although there is a strong association between EBV infection and MS, it is crucial to understand that the development of MS is not solely attributed to EBV infection. Other factors, such as genetics, also significantly contribute to the development of MS.^4^

Acute and past infections of EBV can usually be differentiated based on the presence of specific antibodies. In acute infection, viral capsid antigen (VCA) IgM and VCA IgG without EBNA‐1 IgG are observed. On the other hand, in past infections, the presence of VCA IgG and EBNA‐1 IgG without VCA IgM is commonly seen.^28^ False positives and negatives frequently accompany EBV IgM detection using enzyme immunoassays. There is a limited amount of accuracy in serological markers used to differentiate between acute and past infections with EBV. It is expected to obtain false positives and negatives when detecting EBV IgM, and the determination of EBNA‐1 antibodies presents its challenges. In immunosuppressed patients, secondary loss of EBNA‐1 antibodies is commonly observed. If EBNA‐1 IgG, VCA IgM, and VCA IgG are negative, then it is likely that EBV has been acutely or previously infected and EBNA‐1 IgG has been lost secondary to the infection. When using immunofluorescence assays, the presence of anti‐cellular antibodies may complicate the evaluation of test results. Aside from the false negative results, false positive results, diminished levels, and hard‐to‐interpret results, the tests might be expensive and unavailable for a broad range of individuals in underdeveloped areas.^29^ Table 1 summarizes selected studies on the association between EBV and MS.

**Table 1:** Summary of the selected cohort, case‐control, and cross‐sectional studies on the association between EBV and MS.

Authors/Year/Country	Study design	Follow‐up	MS patients (*n*)/F:M/MS type	PwMS age: Mean (SD)	EDSS: Mean (SD)	DD: Mean (SD)	Controls (*n*)/F:M/Age	Main outcomes
Domínguez‐Mozo et al./2022/Spain^30^	Cohort	2 year	325/ 217:108/ RRMS:325	36 (30−42)^a^	2(1−3)^a^	65.0 m (18−118)^a^	295/ 115:180/ 39 (29.5−47)^a^	MS rarely occurs in the absence of EBV. The relationship between genetic burden and lower EBNA‐1 IgG titers is interestingly linked to an earlier age of MS onset.
Bjornevik et al./2022/USA^1^	Cohort	20 year	955/ 643:312/ NR	NR	NR	NR	1566/ NR/ NR	Risk of MS was increased 32‐fold following infection with EBV, while no increased risk was observed after infection with other viruses. EBV is a major contributor to the development of MS.
Abrahamyan et al./2020/Germany^31^	Cohort	2 year	901/ 630:271/ RRMS: 521 CIS: 380	33 (27−41)^b^	1.5(1−2)^b^	NR	16163/ NR/ NR	Negative EBV serology in patients with suspected inflammatory CNS disease indicates the need to consider alternative diagnoses besides MS.
Munger et al./2019/USA^32^	Case‐control	8 year	170/ 145:25/ NR	19.5 (3.1)	NR	NR	311/ 211:100/ NR	Offspring of women with the highest levels of EBV VCA IgG antibodies during pregnancy have a 2.5 times higher risk of developing MS.
Wergeland et al./2016/Norway^33^	Cohort	2 month	90/ NR/ RRMS: 90	18−55^c^	≤5.5	NR	‐	There are differences in EBNA‐1 IgG levels monthly, an association between EBNA‐1 IgG, 25(OH)D levels, and HLA‐DRB1*15; therefore, EBNA‐1 IgG serum levels are influenced by genetic and environmental factors that impact the risk of MS.
Jakimovski et al./2019/USA^34^	Case‐control	NR	101/ 75:26/ RRMS:69 SPMS:32	46.9 (10.3)	2.5 (1.5−5)^b^	13.3 (10.9)	41/ 29:12/ 45.4 (12.6)	Greater EBV humoral response is associated with lower gray matter magnetization transfer ratio changes and focal destructive lesion pathology in RRMS patients.
Alanazi et al./2022/Saudi Arabia^35^	Cross‐sectional	5 year	1176/784:392/NR	33 (10.77)	4 (0−10) η	NR	NR	EBV is more prevalent among female pwMS than male pwMS (27:1).
Varvatsi et al./2021/Cyprus^36^	Cohort	NR	30/18:12/SPMS:3RRMS:27	46.29 (15)	2.51 (1.97)	NR	33/ 19:14/ 44.37 (12)	EBV genetic variants have a strong association with MS, providing more evidence for potential molecular pathways by which EBV could contribute to the development of the disease.
Bistro et al./2021/Sweden^37^	Case‐control	NR	670/ 563:107/ RRMS: 670	25 (21−29)	NR	NR	670/ 563:107/ 25 (21−29)^b^	EBV infection after adolescence and HHV‐6A infection at any age increase the risk of developing MS.
Hedstrom et al./2020/Sweden^38^	Case‐control	10 year	5289/3910:1379 /NR	16−70^c^	NR	NR	5431/ 4199:1232/ NR	Elevated anti‐EBNA‐1 antibody levels and IM history are different risk factors for MS. The two aspects of EBV infection act synergistically to increase MS risk, and they are involved in the same biological pathways.
Mouhieddine et al./2015/Lebanon^39^	Case‐control	2 year	249/ 148:101/RRMS:196	37.8 (12.1)	2(1.7)	5.9(7.1)	230/ 145:84/ 46.4 (12.5)	An older age and female gender were associated with a higher anti‐VCA titer, while a male gender was associated with a higher anti‐EBNA‐1 titer in pwMS patients.

*Note*: EBV seropositivity refers to the presence of antibodies to EBV in the blood. The association column refers to whether the study found a statistically significant association between EBV seropositivity and MS.

Abbreviations: CIS, Clinically isolated syndrome; CNS, central nervous system; DD, Disease duration; EBNA‐1, Epstein‐Barr nuclear antigen 1; EBV, Epstein‐Barr virus; F, Female; y, year(s); HHV, Human herpesvirus; IgG, Immunoglobulin G; m, month(s); MS, multiple sclerosis; NR, Not reported; PwMS, People with multiple sclerosis; RRMS, Relapsing‐remitting MS; SPMS, Secondary progressive MS; VCA, Viral capsid antigen.

^a^ Median (range).

^b^ Median (IQR), η Mean (range).

^c^ Range.

Multiple studies have reported significantly elevated levels of antibodies against EBV in patients diagnosed with MS compared to healthy individuals serving as controls.^40^, ^41^, ^42^ The EBV can produce specific antibodies and nonspecific antibodies called heterophile antibodies. These heterophile antibodies are often found in high levels in cases of acute IM (AIM), making them a commonly observed biological marker of the disease.^43^ Heterophile antibodies, first identified by Bunnell and Paul, can be found in around 90% of individuals infected with EBV.^44^ The Epstein‐Barr virus nuclear antigen‐1 (EBNA‐1) antibody levels were assumed to reflect levels before MS emerged. Studies indicate that EBNA‐1 antibody levels increase 5−20 years before MS onset and stay constant throughout life.^9^, ^45^ Thus, EBV infection in MS patients is almost universal. Other herpesvirus infections and hepatitis B are less prevalent.^46^ Individuals infected with EBV and have a history of IM or high levels of antibodies against EBV nuclear antigens are more likely to develop MS.^47^ Several studies have discovered that EBNA‐1 antibody titers have been found to interact with HLA‐DRB1*1501, which is the most significant and consistent genetic risk factor for MS.^3^, ^48^ EBNA‐2, an EBV viral protein known for its role in the growth transformation of B cells,^49^ exhibits a higher‐than‐expected frequency of binding to genetic sites associated with the risk of MS.

Furthermore, it was claimed that EBV infection in early childhood is asymptomatic, while in adolescence or young adulthood, it causes several diseases, such as IM. IM is an immunological response defined by a self‐limited lymphoproliferative illness.^50^, ^51^ Hospitalization for IM might increase the risk of MS onset; therefore, IM and MS are strongly associated.^52^

It is believed that EBV‐infected B cells may be involved in MS due to the presence of a high EBV‐IgG index in the cerebrospinal fluid (CSF).^53^ Elevated IgG levels have a strong role in the pathogenesis of MS.^54^ As another piece of evidence, Dooley et al. claimed that EBV latent proteins are common in MS patients' brains, but BFRF1, which is involved in the early lytic cycle of EBV, can only be found in meninges and acute lesions.^55^

**Figure 1:**
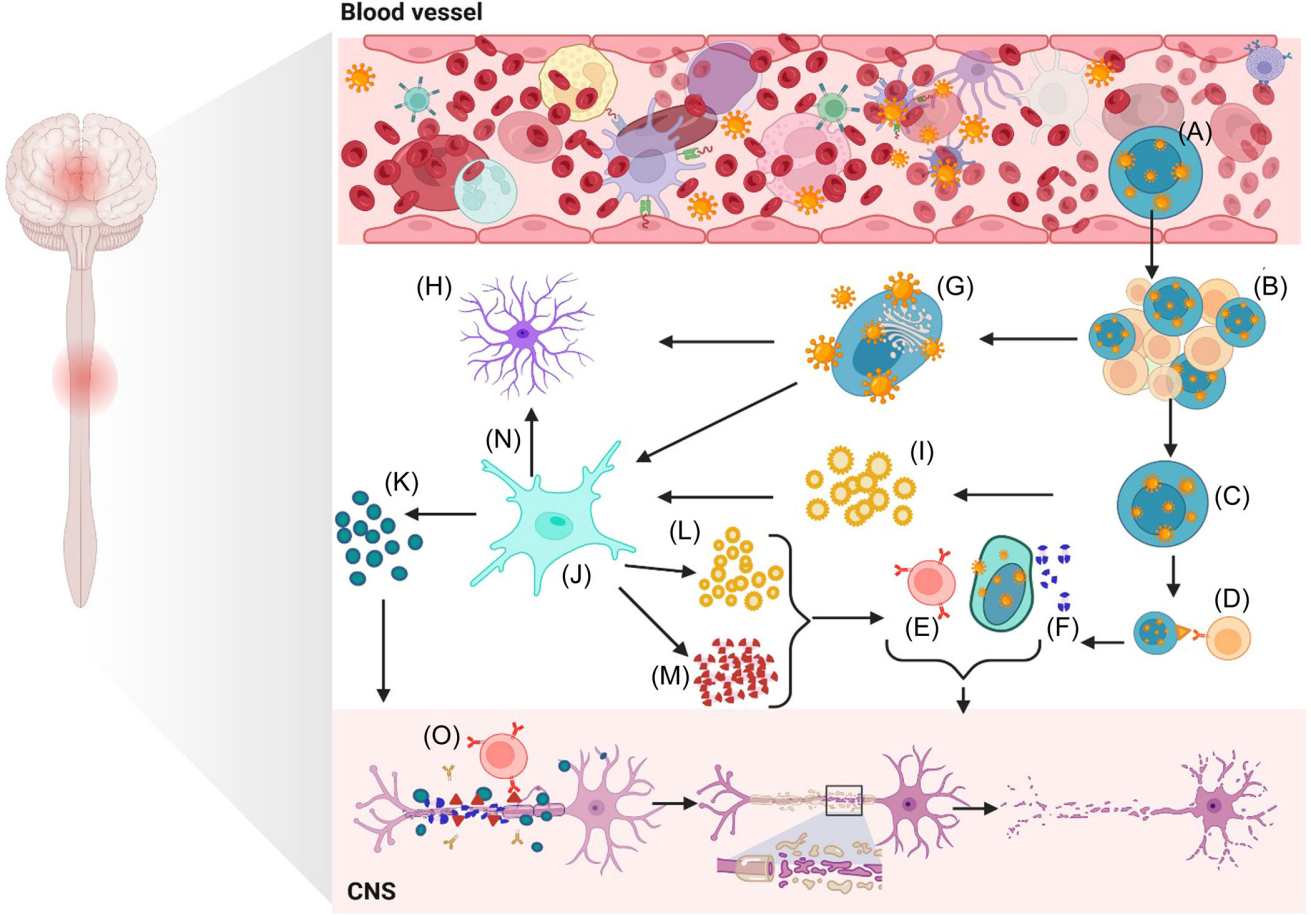
The mechanisms of EBV in MS patients. (A) The EBV virus infects B cells and can establish latent infection in memory B cells. (B) Memory B cells that are infected can develop germinal centers, which then lead to the production of autoreactive B cells. (C) When EBV reactivates in memory B cells, infectious virus particles are produced. (D) Antigen‐presenting cells (APCs) can present infected B cells antigens to autoreactive CD8+ T cells. (E) Activated CD8+ T cells can recognize and kill EBV‐infected B cells, but they may also cross‐react with myelin antigens, causing demyelination. (F) Myelin can be destroyed by antibodies produced by autoreactive B cells. (G) When EBV infects B cells, infectious virus particles are released. (H) Astrocytes can be infected by EBV, leading to neuroinflammation and demyelination. (I) The EBV‐encoded RNAs (EBERs) can be released by infected cells in exosomes and may cause inflammation and demyelination. (J) Inflammation and demyelination can be caused by activated microglia. (K) Activated microglia produce nitric oxide (NO−), which can result in inflammation and demyelination of the neurons. (L) MicroRNAs (miRNAs) are released in exosomes and may contribute to inflammation and demyelination. (M) IFN‐alpha (IFNα) can be produced in response to EBV infection and may contribute to neuroinflammation and demyelination. (N) Neuroinflammation and demyelination can result from activated astrocytes. (O) Autoreactive T cells and B cells may target myelin basic protein/galactocerebroside (MBP/GDP), causing demyelination. [Created with BioRender.com]. EBF, Epstein‐Barr virus infection; INF, increased interferon; MS, multiple sclerosis.

Relapsing‐remitting MS (RRMS) and secondary progressive MS (SPMS) occur when EBV transforms B cells, proliferates in peripheral tissue, and enters the CNS. White matter (WM) lesions recovered T cells, specifically CD8+ T cells, do not recognize CNS autoantigens such as myelin basic protein (MBP). However, they can recognize autologous EBV‐transformed B cells.^56^ Memory B cells infected with EBV will be stimulated by cognate antigens and CD4+ T cells, which leads to the activation of CD8+ cytotoxic T cells. A B cell infected with EBV will irritate T helper and cytotoxic T cells. There seems to be a significant role for T cells in MS lesions based on the predominance of T cells in lesions (Figure 1).^10^ Studies have shown that B‐cell lymphoblastic cell lines (LCLs), produced by EBV transforming B‐lymphocytes in peripheral blood lymphocytes, effectively suppress CD8+ T‐cell responses in MS patients. Moreover, they also regulate IgG‐secreting B cells after EBV infection in vitro. In light of these findings, LCLs may provide a promising therapeutic approach for treating MS, a chronic autoimmune disease that affects the CNS. LCLs may aid in alleviating the symptoms of the disease and possibly halt its progression by reducing the activity of CD8+ T cells.^57^, ^58^

Another investigation reported that CD8+ T cells increased interferon (IFN)‐γ production significantly in response to LCLs in MS.^59^ Various peptides are expressed at different phases of EBV activity at different times. Hence, it is plausible that these discrepancies are attributable to the timing of collection. The range of proteins CD8+ T cells recognizes as antigens of EBV varies with the phase of the infection. The lytic cycle of EBV is characterized by the expression of nuclear (EBNA3A and EBNA3C) and membrane proteins (LMP1 and LMP2) during latency and BZLF1, BRLF1, BMLF1, BMRF1, and BALF4 proteins during early, late, and immediate phases.^60^ By bypassing the antigen processing step shown in normal physiological conditions, it is possible to detect which stages of EBV infection are most vulnerable to peptide attack.^61^

## 4 FOUR HYPOTHESES ABOUT THE CAUSE OF MS BY EBV

### 4.1 The cross‐reactivity of EBV and the molecular mimicry

Pathogenic antigens mimicking host proteins, such as MBP, trigger an immunological response to prevent the immune system from recognizing them as pathogens. This is called Molecular Mimicry.^61^ Among pathogenic processes in immune‐mediated illnesses, an immune response to infectious pathogens may cross‐react with self‐antigens.^62^

Cross‐reactivity has been the most popular hypothesis for many years. It is hypothesized that T lymphocytes previously exposed to EBV can cross‐react with and target antigens found in CNS. Further, when EBV enters the CNS, cross‐reactivity occurs between T cells and B cells infected with EBV. It is possible that the cross‐reactivity of T cells between EBV and CNS antigens had a role in MS development. However, it's not very probable to consider it a fundamental cause.^8^

EBV antigens can trigger reactive T‐cell clones associated with MS.^63^ Defective removal of EBV‐infected B cells by CD8+ T cells may increase the risk of MS by allowing the buildup of EBV‐infected self‐reactive B cells in CNS.^64^ Recent research suggests that LCLs do not cross‐react with brain antigens. However, there is evidence that a small percentage (3−4%) of CD4+ T lymphocytes specific to EBNA‐1 in healthy donors and MS patients interact with myelin basic protein (MBP) peptides.^57^, ^65^ In conclusion, this hypothesis suggests that the similarities between EBV antigens and the surface proteins of hosts' cells initiate the autoimmunity cascade.

### 4.2 The hypothesis of bystander damage

MS is not considered an autoimmune disease according to this theory. However, secondary autoimmune reactions may occur when the CNS is exposed to antigens produced after viral‐targeted bystander injury. The strong association between CD8+ T lymphocytes and EBV‐infected plasma cells in the brain supports this notion. RRMS is related to clinical relapses as well as gadolinium‐enhancing MRI brain lesions. These symptoms indicate an increase in the number of CD8 + T cells specific for EBV lytic antigens in the blood.^66^

Clinical attacks of MS typically exhibit inflammation, demyelination, and neurological impairments. However, the cause of those symptoms is uncertain, whether it is due to EBV‐specific CD8+ T cells or CNS‐reactive T cells.

The primary inquiry is why the immune response targeting EBV fails to eliminate the infected B cells with EBV, despite causing damage to CNS of bystanders. One research consistently identified that active EBV infection in B cells infiltrates MS patients' brains with secondary progressive or acute disease (nearly in all patients).^7^

EBV‐directed bystander damage may be associated with the progression of MS. However, it is unlikely to constitute EBV's primary pathogenic function in MS.^8^ Some others claimed that EBER molecules generated by EBV‐infected B cells may cause inflammation in the CNS.^67^ In summary, according to this hypothesis, postviral CNS damage eventually results in inflammation and the subsequent appearance of MS symptoms.

### 4.3 αB‐Crystallin or “mistaken self” hypothesis

Immunodominant B‐Crystallin expression was reported in the myelin and oligodendrocytes of MS patients despite normal WM. According to the theory, applying a stimulus can increase αB‐Crystallin levels in lymphoid cells (invading the CNS) and oligodendrocytes. EBV can stimulate the expression of αB‐Crystallin in B cells, which is subsequently absorbed by CD4+ T cells. Thus, this approach may explain the link between CD4+ T cell‐targeted oligodendrocytes and myelin I.^61^ It remains a hypothesis due to the lack of strong evidence to support it.

### 4.4 The hypothesis that EBV‐infected B cells produce autoreactive antibodies

Cyto‐reactivating autoreactive B cells in a particular organ causes EBV to move and accumulate antibodies in these organs. Consequently, infectious agents produce and cross‐reactivate pathogenic autoantibodies. Auto‐reactive CD4+ T cells prevent apoptosis by providing stimulated survival signals with infected B cells. Auto‐reactive T cells can produce cytokines, proliferate, and absorb cells, eventually causing damage by targeting organs and chronic autoimmune diseases.^64^ This idea is advantageous because it shows a connection between B cell biology and EBV.^68^

Furthermore, the presence of EBV‐encoded molecules (LMP1 and LMP2) in EBV‐infected cells during a germinal center reaction may facilitate the survival and entry of “forbidden” autoreactive B cells into the memory B cell pool. Even though they normally wouldn't receive survival signals from cognate antigens and T helper cells.^68^ Another research examined recombinant antibodies extracted from single EBV‐positive and EBV‐negative memory B cells. They showed that although EBV still persists in self‐ and polyreactive B cells, EBV‐positive memory B cells have a lower occurrence of self‐ and polyreactive autoantibodies than EBV‐negative memory B cells. Also, they reported that although EBV persists in self‐ and polyreactive B cells, there is no evidence that EBV facilitates the survival of “forbidden” autoreactive B cells.^69^

MS patients have reported a beneficial response to anti CD‐20 therapy such as ocrelizumab, ofatumumab, and rituximab in correlation with reduced B cells. Rituximab in primary progressive MS (PPMS) has been shown to specifically reduce B cells in peripheral blood and CSF, and IFN‐β decreases pathogenic memory B cells. The growing usage of monoclonal agents that effectively target B cell population depletion in MS serves as further evidence for the significant contribution of B cells to the development of MS. Consequently, identifying the underlying factors responsible for the abnormal B cell activity in MS is expected to be a critical step in comprehending the etiology of MS.^61^

### 4.5 EBV infection can alter the immune system functions

In addition, EBV may alter the effector and regulatory T‐cell balance, B‐lymphocyte growth, and permeability of the blood‐brain barrier (BBB).^70^ As a result of inflammation, the BBB can be breached, allowing lymphocytes to invade the brain. It has been hypothesized that EBV+ or EBV‐specific B cells may exacerbate MS plaques through constant inflammatory processes, EBV reactivation, and/or molecular mimicry once they become resident on the MS plaque.^71^

## 5 CONTROLLING EBV INFECTION TO PREVENT AND TREAT MS

It may be possible to treat MS with therapeutic vaccines or antiviral drugs that target EBV.

### 5.1 Antiviral compounds

Herpes zoster, shingles, chickenpox, and genital herpes are effectively treated with Famciclovir and Acyclovir.^72^ Acyclovir's activity against EBV is much less than that against herpes simplex virus (HSV) and varicella‐zoster virus. Using acyclovir to treat EBV‐associated IM does not appear to be efficacious enough. On the other hand, it seems to reduce virus transmission. Unlike Acyclovir, Ganciclovir (dihydroxy‐ propoxymethylguanine) is more effective at combating EBV, but it is also harmful, which makes its use in otherwise healthy adults challenging to justify.^73^ Viral thymidine kinase enzyme is not expressed in lymphoid disorders (in contrast to lytic diseases), so neither drug is a good choice. Combination therapy with arginine butyrate and Ganciclovir for EBV‐associated lymphoproliferation after solid organ transplantation seemed to be effective.^74^

In comparison with the standard drug ganciclovir, valganciclovir is a prodrug that has a much longer half‐life and is more bioavailable. Active EBV infections have been successfully treated with this medication. Approximately 75% of patients with chronic active EBV infection responded to valganciclovir in a clinical trial.^75^

A cytokine known as pegylated IFN‐α‐2a has functions that are both antiviral and immunomodulatory. The drug has been used for the treatment of diseases associated with chronic active EBV. There is evidence that IFN‐α can induce remission in some patients in a pilot study.^76^

In the absence of viral thymidine kinase, Foscarnet can inhibit viral DNA polymerase.^77^ Human IFN‐β, a potent antiviral protein, is among the top five MS treatments. IFN‐β may have an anti‐inflammatory effect despite its uncertain mode of action in MS. It was found that IFN‐β inhibited EBV, CMV, and other viruses' ability to replicate and influenced T cell proliferative responses to EBNA1 and decreased memory B cells, which are linked to MS and considered pathogenic cells.^78^

However, new evidence shows that some antiviral drugs and treatment techniques (such as adoptive immunotherapy using cytotoxic T cells or B cells targeting EBV and delivery of specific monoclonal antibodies) may offer great potential for treating EBV‐associated malignancies.^79^ In addition, infusion of EBV‐specific T cells,^80^ histone deacetylase (HDAC) inhibitors (It is widely known that 11 HDAC isoforms inhibited by these drugs induce the lytic phase of EBV),^81^ and EBNA‐1 inhibitor.^82^ These treatment strategies can reduce the antigenic stimulus that stimulates EBV, specific antibodies, and T cells to damage the nervous system.^83^

A major drawback of this treatment, however, is that EBV load is not generally increased in people with MS, so reducing virus‐infected cells may be ineffective, and prevention is a better approach to control this virus.^84^

### 5.2 Vaccination

A vaccine approach would be the most effective way to prevent EBV‐associated diseases, similar to other infectious agents; it would prevent infection and induce sterilizing immunity.^85^

A new advancement in vaccination that uses genomic vaccines capable of delivering various protein sequences may lead to more effective vaccination experiments in MS.^86^ Nonetheless, the potential of creating a vaccine that might block or prevent EBV infection from preventing the onset of MS remains alluring but riddled with obstacles. Developing a vaccine against EBV is problematic because it is almost impossible to achieve sterile immunity against any herpesvirus. A vaccination that prevents AIM might reduce the incidence of MS.^87^

Over the last four decades, EBV vaccines have been advocated, but none have been approved. There is a lack of adequate animal models for EBV diseases, except for nonhuman primates; Herpesvirus vaccines are feared to be oncogenic and are believed to be uncommercial viable because of concerns about their oncogenic capacity.^88^

Gu et al. reported the phase 1 trial results of the gp220–340 vaccinations in 1995. The immunogenicity of the vaccine was evaluated in rabbits, and subsequently, its safety was assessed in 11 adults and six children with a history of EBV infection.^89^

The next phase included immunizing 19 EBV‐unaware youngsters between the ages of 1.7−2.8. Ten youngsters served as controls, and nine were satisfied with a single vaccination dosage. Within 6 months, eight of nine vaccine recipients developed antibodies against gp220−340. All 10 participants in the control group and three of the nine vaccine recipients became immune to the wild‐type EBV VCA, which is not present in the vaccine. No more study has been recorded despite the vaccine's apparent efficacy; this is likely because it includes live vaccinia, which has been associated with negative impacts.^90^

### 5.3 Inclusion of soluble EBVgp350 in subunit vaccines

Because of the close relationship between antibodies against EBVgp350 and neutralizing antibodies, using EBV membrane antigen (containing gp350) as the immunogen makes sense.^88^

A potential preventive measure against AIM in young adults who were born to seronegative carriers of EBV is the administration of the recombinant gp350 vaccine. Vaccinating with gp350 may lower the likelihood of MS by reducing AIM, which is associated with an increased risk of MS. Vaccination does not provide protection against asymptomatic EBV infection. Seroconversion to anti‐gp350 antibodies occurred after vaccination and lasted for 418 months, resulting in the protective effect since anti‐gp350 antibodies neutralize EBV infectivity.^91^ It is possible to administer vaccinations to infants after passively transmitted antibodies from their mothers have waned, typically around 6−9 months of age. Providing early protection against EBV would reduce the risk of developing EBV‐related diseases later in life. The immune response to EBV may wane over time, requiring repeated boosters during life to avoid lytic infection.^92^

Studies show that Rhesus monkeys immunized with soluble rhesus lymphocryptoviral gp350 not only had greater resistance to infection but also reduced viral loads after exposure.^93^ In the Phase I/II study, EBV‐naive volunteers aged 18−37 were randomized into three groups: unadjuvanted vaccine, AS04‐adjuvanted vaccine, and aluminum salt‐adjuvanted vaccine. Data on the immunogenicity of the vaccine by measuring neutralizing antibodies and gp350 showed that vaccines adjuvanted with AS04 or aluminum salt performed better than those without adjuvants. As part of the third trial, phase 2 placebo‐controlled, double‐blind studies were conducted in EBV‐naive Belgians 16–25 years of age to assess the safety, immunogenicity, and efficacy of the recombinant gp350 vaccine adjuvanted with ASO4. Vaccines were administered intramuscularly at 0, 1, and 5 months. 76/77 (96.7%) of individuals who received the vaccination and were not afterward infected with wild‐type EBV developed antibodies against gp350. The vaccination did not prevent infection; 13 vaccine recipients (14%) and 18 placebo participants (20%) contracted the disease.

Nonetheless, it substantially impacted the patient's clinical picture.^94^ Lastly, 16 pediatric kidney transplant candidates were enrolled in a phase 1 study of a recombinant gp350 vaccine with aluminum hydroxide adjuvant. GP350 was well tolerated when given subcutaneously three to four times over 32 weeks. Only 4 out of 13 vaccines produced neutralizing antibodies against gp350.

### 5.4 CD8 + T‐cell peptide epitope vaccine

In a patient with SPMS reported by Pender et al. it has been hypothesized that the increase of CD8+ EBV reactive T cells may alleviate symptoms of this disease. According to the results of the study, while EBV‐reactive CD8+ T cells increased in this study, gadolinium MRI brain lesions decreased, and levels of CSF IgG significantly reduced from 0.79 to 0.63.^95^ In 2015, this group began a clinical trial in which in vitro‐expanded autologous EBV‐specific T cells were applied to 10 patients with PPMS and SPMS. Based on the results of the study, the EBV‐targeted T cell therapy was well tolerated and resulted in clinical improvements, which was attributed to the effective response of the administered T cells to the EBV virus.^96^

This strategy is to generate CD8+ T‐cell immunity to EBNAs to limit the expansion of EBV‐infected B cells. Ex vivo peptide‐specific IFN‐γ production demonstrated that this strategy successfully produced peptide‐specific CD8+ T cells in most individuals. It was found that one of the two placebo recipients who later became infected with wild‐type EBV developed IM, whereas none of the four vaccinated individuals had symptoms. Epitope vaccines are designed to target specific HLA types, so their general utility is limited. In transplant patients whose HLA status was established before the transplant, epitope vaccinations may still help avoid PTLD.^97^

### 5.5 Prospects for future prophylactic EBV vaccines

As a result, molecular mimicry, where antibodies or T cells to EBV proteins react with CNS proteins, is suggested as a potential explanation for the link between EBV and MS. If this hypothesis is true, immunizing with specific parts of EBV proteins that react with CNS proteins, like EBNA‐1, could lead to an immune response that raises the risk of MS. Therefore, it is important to carefully decide which viral antigens should be included in EBV vaccines.^47^ A preventive EBV vaccine can reduce the impact of various diseases caused by EBV. It would be sufficient, even if a candidate vaccine only prevented or modified IM. There is a compelling reason to make this a pediatric vaccine since it had previously only been prescribed for school entry. This vaccine's effectiveness is highly variable across races and ethnicities. Given that IM stems from primary EBV infection and poses a potential risk for Leukemia and MS, expediting vaccinations appears prudent. MS may potentially be avoided by receiving an EBV latent protein vaccine.^87^, ^98^ Major challenges in developing an EBV vaccine include a lack of knowledge about the specific factors that provide protection, uncertainty regarding the choice of adjuvants, and the absence of suitable animal models for testing.^99^ To evaluate the impact of the EBV vaccine on MS, it is necessary to conduct national registry studies that track the occurrence of MS in both vaccinated and unvaccinated individuals. This will help determine if the vaccine has any effect on MS. However, assessing the efficacy of the vaccine poses several challenges, as MS is a delayed consequence of EBV infection, typically appearing around the age of 30.^100^ No licensed EBV vaccine exists currently. To advance the development of EBV vaccines for MS prevention, a deeper understanding of the connection between EBV and MS is necessary.^101^

## 6 CONCLUSION

In conclusion, several attempts have been devoted to comprehending the mechanisms contributing to EBV infection and MS risk, including molecular mimicry and an altered immune response to EBV infection. Nevertheless, a complete understanding of these mechanisms remains elusive. Considering its infectious nature and ability to escape the immune system, EBV may be a strong contender for being the cause of MS. Our review suggests the role of EBV in the pathogenesis of MS must not be underestimated as there are robust physiological mechanisms giving rise to MS‐related demyelination in EBV‐infected individuals. Nevertheless, further research is required to elucidate the exact details underpinning this process.

Developing a vaccine for EBV could be beneficial in preventing MS in these patients, but creating an effective and safe vaccine will be a major hurdle. Although there are many challenges in producing a vaccine against EBV, many scientists are working on this project, which may lead to unexpected outcomes. Studies on various herpesvirus vaccines have shown promising findings, lending credence to the possibility of creating a vaccination that would protect against illness rather than infection.

## AUTHOR CONTRIBUTIONS

**Mahtab Mohammadzamani**: Conceptualization; data curation; supervision; validation. **Kimia Kazemzadeh**: Conceptualization; data curation; validation; writing—review and editing. **Swati Chand**: Writing—original draft; writing—review and editing. **Sangharsha Thapa**: Supervision; visualization; writing—original draft; writing—review and editing. **Narges Ebrahimi**: Conceptualization; data curation; writing—original draft; writing—review and editing. **Mohammad Yazdan Panah**: Validation; writing—review and editing. **Vahid Shaygannejad**: Supervision; validation. **Omid Mirmosayyeb**: Supervision; validation; visualization. The corresponding author confirmed that all of the authors have read and approved the final version of the manuscript.

## CONFLICT OF INTEREST STATEMENT

The authors declare no conflict of interest.

## TRANSPARENCY STATEMENT

The lead author Sangharsha Thapa affirms that this manuscript is an honest, accurate, and transparent account of the study being reported; that no important aspects of the study have been omitted; and that any discrepancies from the study as planned (and, if relevant, registered) have been explained.

## Data Availability

Data available on request from the authors. The corresponding author had full access to all of the data in this study and took complete responsibility for the integrity of the data and the accuracy of the data analysis.
